# Inpainting for Saturation Artifacts in Optical Coherence Tomography Using Dictionary-Based Sparse Representation

**DOI:** 10.1109/jphot.2021.3056574

**Published:** 2021-02-02

**Authors:** Hongshan Liu, Shengting Cao, Yuye Ling, Yu Gan

**Affiliations:** 1 Department of Electrical and Computer Engineering, The University of Alabama, Tuscaloosa, AL 35487 USA; 2 John Hopcroft Center for Computer Science, Shanghai Jiao Tong University, Shanghai 200240, China

**Keywords:** Inpainting, optical coherence tomography, saturation artifacts, sparse representation

## Abstract

Saturation artifacts in optical coherence tomography (OCT) occur when received signal exceeds the dynamic range of spectrometer. Saturation artifact shows a streaking pattern and could impact the quality of OCT images, leading to inaccurate medical diagnosis. In this paper, we automatically localize saturation artifacts and propose an artifact correction method via inpainting. We adopt a dictionary-based sparse representation scheme for inpainting. Experimental results demonstrate that, in both case of synthetic artifacts and real artifacts, our method outperforms interpolation method and Euler’s elastica method in both qualitative and quantitative results. The generic dictionary offers similar image quality when applied to tissue samples which are excluded from dictionary training. This method may have the potential to be widely used in a variety of OCT images for the localization and inpainting of the saturation artifacts.

## Graphical Abstract

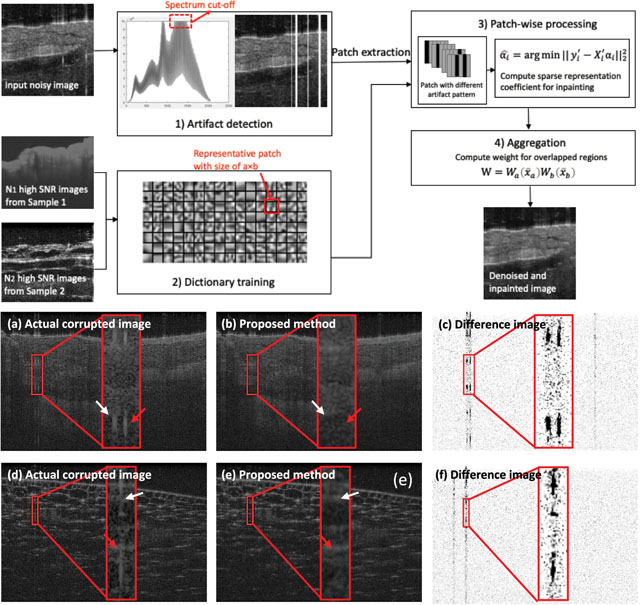

## 1. Introduction

**Fig. 1.**
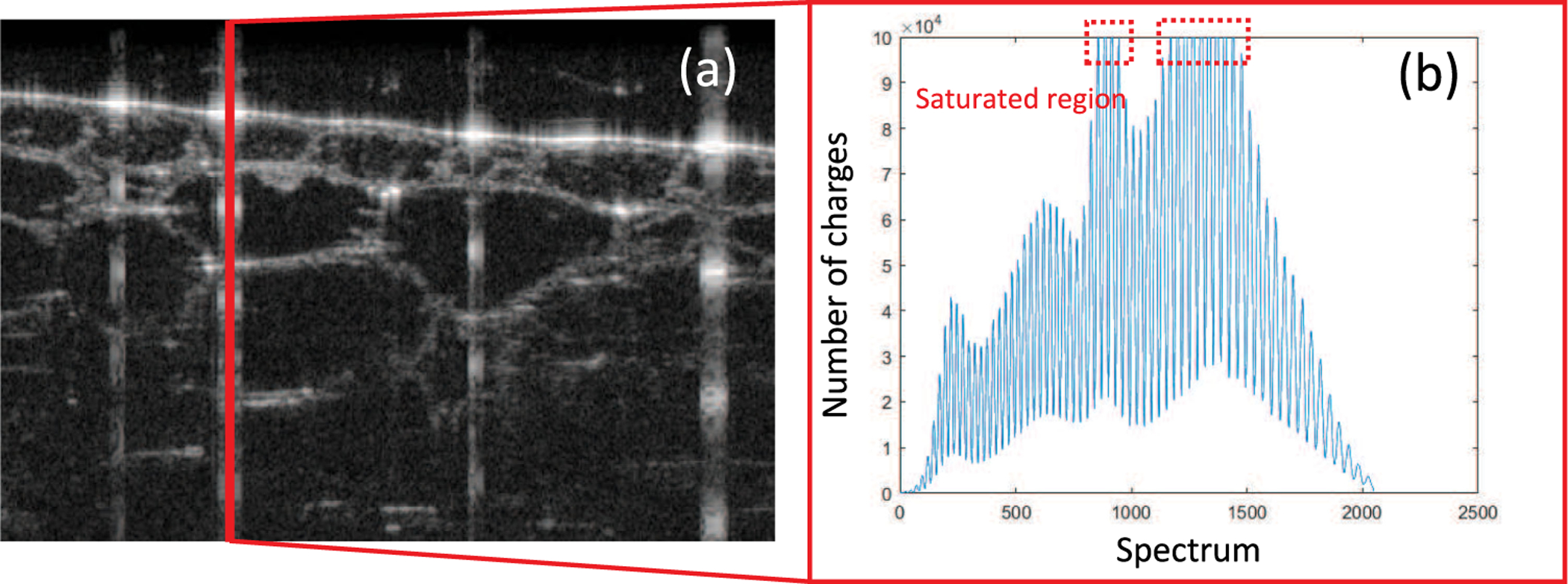
Examples of saturation artifacts in OCT images: (a) B-scan from onion sample; (b) corresponding saturated spectrum.

Saturation artifacts are commonly observed in optical coherence tomography (OCT), which could degrade the segmentation accuracy [1] and lead to errors in derived clinic parameters [2]. Take spectral-domain OCT (SD-OCT) as an example, it uses a linear charge-coupled device (CCD) camera as detector to measure spectral interferogram [3]. A reflectivity profile is then reconstructed along the axial direction, the direction that is parallel to the light propagation, in the form of axial line (A-line). SD-OCT suffers from saturation artifacts. If the detected signal’s fluctuation exceeds the dynamic range of the CCD [4], it will give rise to saturation artifacts that is manifested by extraordinarily bright A-lines in the OCT images as shown in Fig. 1(a). Those artifacts, featured by streaking patterns, are often caused by specular reflections from the interfaces. Importantly, the existence of saturation artifacts could hinder the in-depth analysis of OCT images, such as segmentation.

To reduce the impact of saturation artifacts, various methods have been proposed via improving current software [5], [6], refined image acquisition protocols [7], [8], or innovative hardware design [9]. Linear interpolation was used in [5] to reduce saturation artifacts. However, the performance of such interpolation-based method will degrade when A-lines are densely saturated. In [6], local polynomial model was employed to interpolate distant artifacts to improve the segmentation accuracy of corneal OCT images, but the precise estimation can be obtained only for central artifact region. The distant region (low-SNR) was interpolated by local polynomial model which takes the detected surface as reference, therefore it increased speckle noise pattern. Wu *et al.* proposed an imaging acquisition process using a dual-line CCD as the detector [7], [8]. They compensated saturated points of one line using the signal from the other line. To improve the hardware design, a two-channel detector system was proposed to compensate the saturated points using the signal from the channel in which the signal was not saturated in [9]. Because the ratio of signal on the two channels is non-linear across spectral domain and is roughly estimated, the compensation may lead to high error when the reference signal is highly out-of-focus. Moreover, hardware-based methods introduce additional cost to the implementation and lack adaptability in practical applications. As above interpolation methods produce less satisfactory outcomes when dealing with dense saturation artifacts, we seek to solve this problem via algorithmic efforts, sparse representation.

Sparse representation is related to the compressed sensing (CS) theory; if a signal is considered sparse or compressive, it can be faithfully reconstructed by exploiting a small amount of measurements, which is much less than the one suggested by Shannon’s sampling theorem [10]. We can formulate the correction of saturation artifacts as an inpainting problem, which reconstructs missing values in OCT images from a few known measurements [11]. There are multiple methods to address such inpainting problem. An Euler’s elastica variation image inpainting model [12] can be used to reconstruct the distorted image by minimizing the Euler’s elastica energy function. However, this model involves multiple parameters, and the performance is not satisfactory. For this OCT image inpainting caused by saturation artifacts, we formulate it as a dictionary learning problem in sparse representation. It assumes that each signal can be represented by a linear combination of sparse elements [13] from a pre-specified dictionary. The dictionary is a set of elementary atoms and can be used to decompose signal. The dictionary is built based on a mathematical model and learning [14]. Mathematically defined dictionary, such as the sparsifying transform corresponding to the Fourier transform, is used to reconstruct the OCT image based on the CS theory [15]. Typically, the dictionary is trained based on the representative samples to achieve the better performance [16]. Under the framework of sparse representation, noise cannot be represented efficiently. Therefore noise-free structural information is well preserved after sparse representation process [17]. In OCT community, sparse representation has been used to reduce speckle noise or super-resolve down-sampled images [11], indicating great potential to be used for correcting saturation artifacts as a solution of inpainting using dictionary learning.

In this paper, we propose a novel method to localize and correct saturation artifacts in SD-OCT images. Specifically, we formulate the artifact removal problem as an image inpainting problem and adopt sparse representation framework to solve it. We first localize the saturation on A-line level and generate a mask which indicates the saturated regions. In particular, we train a generalized dictionary which includes OCT images from distinct types of samples. We devise a patch-based approach to perform image inpainting in the saturated region using dictionary-based sparse representation. The feasibility is demonstrated with synthetic artifacts and real artifacts. We further demonstrate that our design can be generalized to the scenario when the testing tissue type was not involved in dictionary training. Our experiment indicates that the proposed method outperforms cubic spline interpolation (SI) and Euler’s elastica method both qualitatively and quantitatively.

## 2. Method

### 2.1 Algorithm

**Fig. 2.**
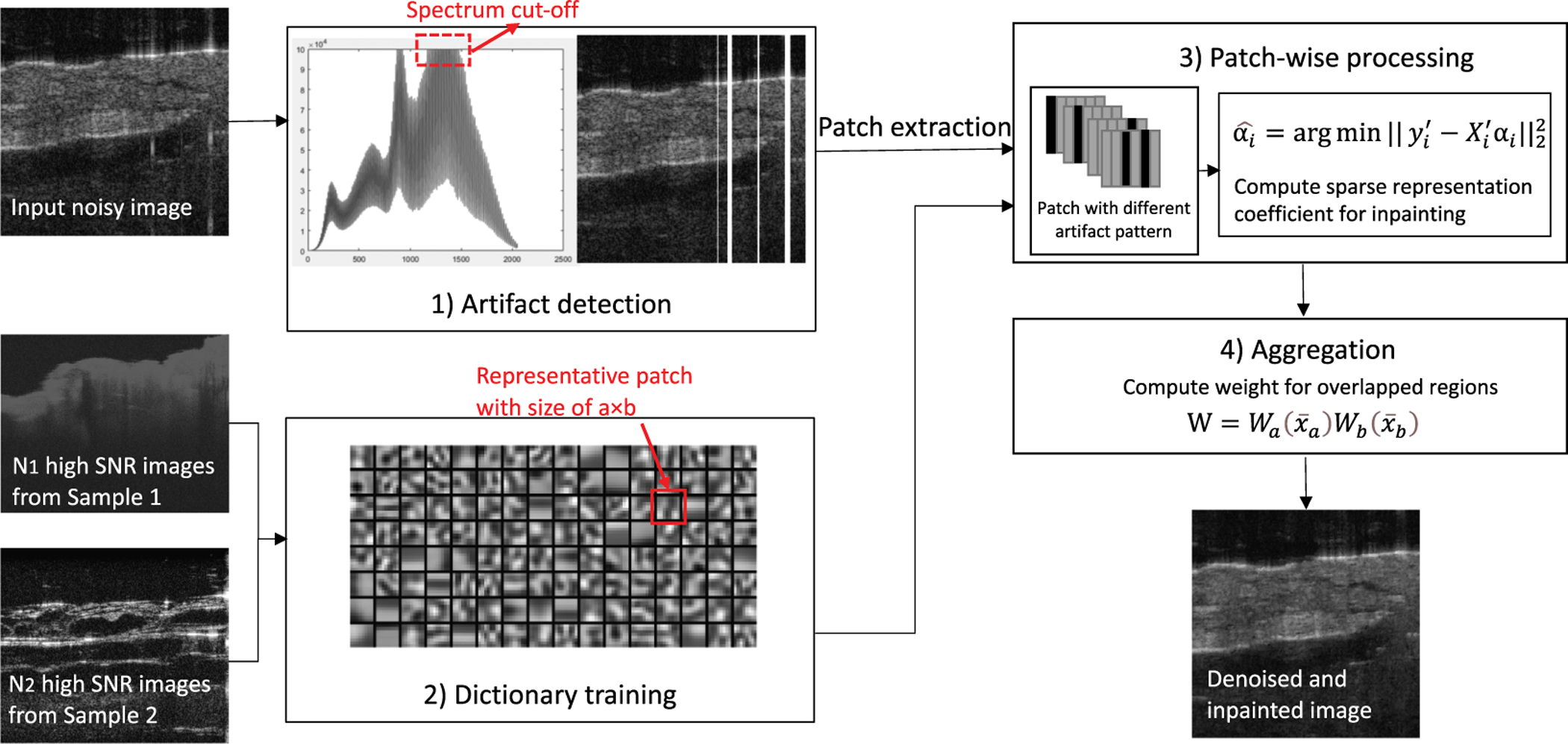
Flowchart of the proposed algorithm: 1) artifact detection for the input noisy image, 2) dictionary training for solving the sparse representation problem, 3) patch-wise processing for inpainting saturated patch, and 4) aggregation.

The proposed method consists of four major modules as Fig. 2 depicts: 1) saturation artifact detection, 2) dictionary training, 3) patch-wise processing, and 4) aggregation.

The input noisy image is first fed into a saturation artifact detector. The original noisy image aligned with detected artifact mask is divided to overlapping patches. The patches with saturation will be processed via a dictionary-based inpainting process to predict the actual pixel value of corrupted region. We train a dictionary using external image set with high signal-to-noise-ratio (SNR). The output image is aggregated by weighted summation.

### 2.2 Artifact Detection

In SD-OCT, CCD, an array of photodetectors, measures interference signals in a range of specific frequency domain [18], [19]. The maximum amount of charges that a single pixel can store is defined as the full well capacity of a spectrometer [20]. Saturation artifacts are caused by strong reflection generated from a highly reflective surface. When the detected signal exceeds the dynamic range of spectrometer in SD-OCT, the pixel will be saturated, and the image will present a bright vertical line [4]. We develop a module to automatically identify the occurrence of saturation in spectral domain prior to image reconstruction.

#### 2.2.1 Saturated Point Detection:

**Fig. 3.**
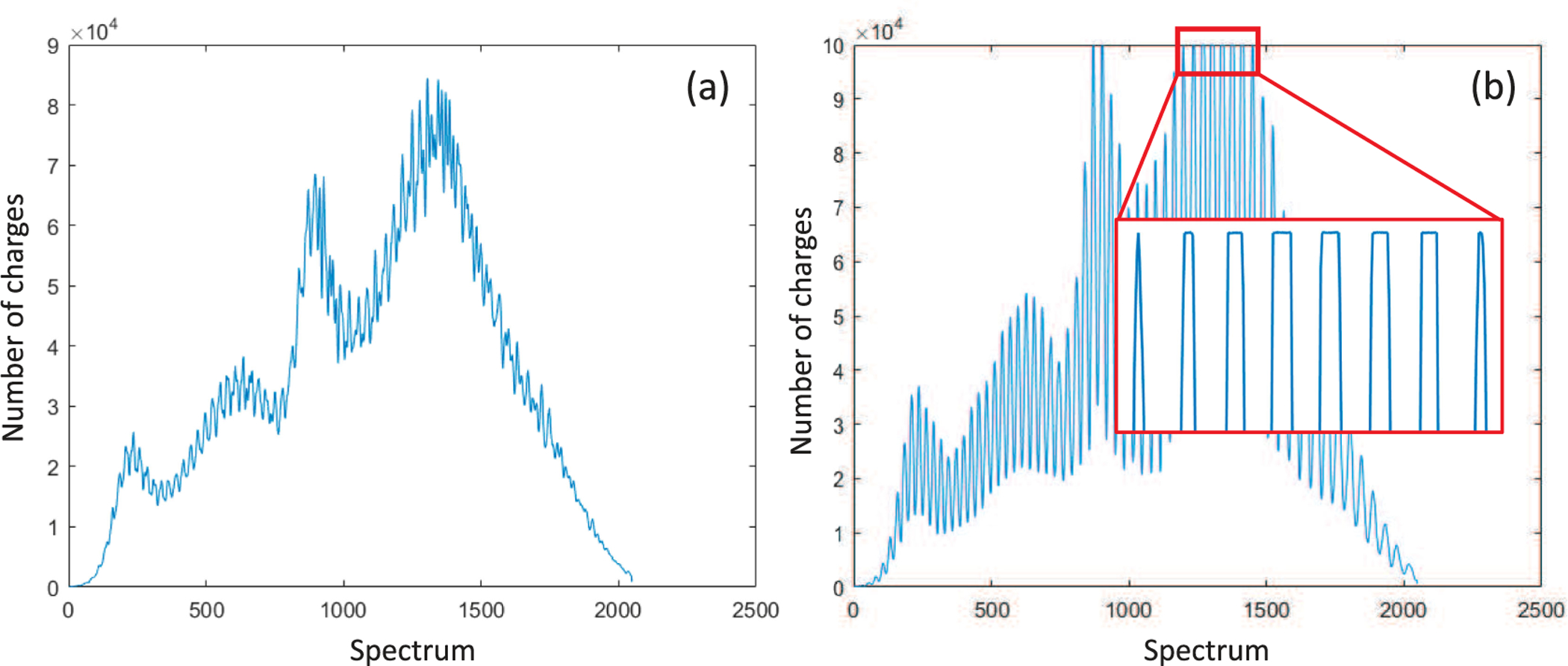
Comparison between (a) unsaturated spectrum and (b) saturated spectrum. Red frame corresponds to cut-off region.

We localize the saturation on A-line level by detecting the number of received charges on each spectrum point. If there is any spectrum point which achieves the full well capacity in an A-line, we label the corresponding A-line as being saturated. We observe that, in an unsaturated A-line (Fig. 3(a)), the maximum number of received charges of a spectrum point is usually less than the full well capacity. Saturated A-line (Fig. 3(b)) has a cut-off region (red frame), which includes several consecutive points reaching the full well capacity in each peak.

#### 2.2.2 Saturation Artifact Mask:

After we examine all A-lines in a B-scan, we output a column-wise mask indicating all saturated A-lines. As shown in Fig. 2, the original OCT image, overlaid with its mask, is sent to patch extraction and processing.

### 2.3 Dictionary Learning

We build a generalized dictionary for inpainting. It is an overcomplete dictionary by using *N* high SNR images obtained by external averaged images as training data in sparse representation. The *N* external averaged images are selected evenly from various types of samples, such as coronary and onion. To make this dictionary applicable to OCT images with different size, we opt to divide OCT images into patches and use a single patch as a unit for dictionary learning and processing. We extract overlapping patches $Y_{\text{train}}\in {\mathbb{R}}^{n}$ with a size of *a* × *b* from training images. We convert each patch to a column vector $y_{\text{train}}\in {\mathbb{R}}^{ab\times 1}$. To normalize each patch, we subtract mean value of intensity from each pixel. We train the overcomplete dictionary ($X\in {\mathbb{R}}^{ab\times q}$, *ab < q*, where *q* represents the number of dictionary atoms) by using K-SVD and batch-OMP algorithm [21] to solve the following problem:

(1) $$\hat{\alpha }=\text{arg min}\|\alpha {\|}_{0}\text{ s.t.}{\left\|y_{\text{train}}-X\alpha \right\|}_{2}^{2}\leq \varepsilon$$

where ‖***α***‖_0_ is the *l*_0_ norm, defined as the number of non-zero elements in vector *α*, *ε* is specific representation error, a constant for all patches, which describes the termination condition. As indicated in Fig. 2, representative patches are small sub-blocks with size of *a* × *b*.

### 2.4 Patch-Wise Processing

Similar to images for dictionary learning, the input image is decomposed into overlapping patches $Y_{i}\in {\mathbb{R}}^{n}$ of size *a* × *b*. Each patch *Y*_*i*_ is then converted to a vector form $y_{i}\in {\mathbb{R}}^{ab\times 1}$ in lexicographic order. To standardize the patches, we subtract the mean intensity value from each pixel. We perform sparse representation on divided patches. In particular, we perform inpainting for patches which contain at least one saturated pixel.

The process of filling the saturated pixels is to solve a sparse-coding problem where the sparse coefficient $\hat{\alpha_{i}}$ is obtained by a subset of pixels (unsaturated pixels within the patch). Considering a case where we have *n* pixels saturated in a patch, we first remove the saturated pixels from vector $y_{i}\in {\mathbb{R}}^{ab\times 1}$ and get the new vector $y_{i}^{\prime }\in {\mathbb{R}}^{(ab-n)\times 1}$. Accordingly, rows with the same index as *n* saturated pixels are also removed from the dictionary $X\in {\mathbb{R}}^{ab\times q}$, resulting a new overcomplete dictionary $X_{i}^{\prime }\in {\mathbb{R}}^{(ab-n)\times q}$. For an inpainting problem, we assume that the remaining pixels are reliable. By decomposing the remaining pixels on a set of elementary atoms in reduced dictionary, we calculate sparse coefficient for the artifact-free patch as

(2) $${\hat{\alpha }}_{i}=\text{arg min}{\left\|y_{i}^{\prime }-X_{i}^{\prime }\alpha_{i}\right\|}_{2}^{2}\text{ s.t.}{\left\|\alpha_{i}\right\|}_{0}\leq p$$

where $y_{i}^{\prime }$ is a vector of unsaturated pixels, $X_{i}^{\prime }$ is corresponding reduced dictionary, and *p* represents the sparsity level. We use K-SVD and batch-OMP algorithm to solve this sparse representation problem. As an inpainting process, we use the sparse coefficient and original dictionary to reconstruct the entire patch which has a size of *ab* × 1 by calculating $\hat{\alpha_{i}}X$. In sparse representation, the inpainted patches are assumed to be well estimated on the remained pixels. As an inverse process of standardization, we restore the mean intensity value and have the estimated vector by $Z_{i}=X\hat{\alpha_{i}}+m_{i}$.

### 2.5 Aggregation

Overlapping patches are extracted prior to sparse representation, therefore every pixel has multiple estimated values from multiple patches. One potential drawback is edge effect, which happens if all patches are simply restored back. The transition across the edge of each patch harms homogeneity of whole image as the magnitude difference is distinct across the edge [22]. Besides, the estimated pixel located at the boundary of patch has less confidence because it takes less advantages of neighboring pixels. Therefore, weighted summation scheme is adopted to generate estimated pixel. In each patch, the pixel in center is assigned a higher weight, while the pixel near the edge is assigned a lower weight. The weight is based on the location of pixel in a patch. For the pixel locates at (${\bar{X}}_{a}$, ${\bar{X}}_{b}$) in a patch, the weight is

(3) $$W=W_{a}\left({\bar{X}}_{a}\right)W_{b}\left({\bar{X}}_{b}\right)$$

where $W_{a}\left({\bar{x}}_{a}\right)$ and $W_{b}\left({\bar{x}}_{b}\right)$ are linear variation from 1 to 0 corresponding to center to edge. The overlapping patches are multiplied by the weight, and then added up to calculate the estimated pixel value in the output image.

## 3. Experimental Setup and Results

### 3.1 Data

To evaluate the performance of our artifact removal method, we use three OCT datasets, including OCT volumes from human coronary, onion, and human finger respectively. Samples are imaged via SDOCT system (Thorlabs Ganymede, Newton, NJ) with an axial resolution of 3 *μ*m and a lateral resolution of 4 *μ*m, all in air. Coronary image is with a size of 1024 × 1500 pixels, corresponding to a space of 1.98 × 3 mm^2^. Onion image is with a size of 750 × 1024 pixels, corresponding to a space of 1.98 × 1.5 mm^2^. Finger image is with a size of 1024× 400 pixels, corresponding to a space of 1.98 × 4 mm^2^. Autopsy specimens of heart vessel are collected and imaged through the same protocol in [23]. All images are acquired in the laboratory at the University of Alabama.

### 3.2 Experiment

#### 3.2.1 Parameter Setting:

The parameters in the following manuscripts are set based on our experiments and suggestions in [11]. We empirically set representation error for dictionary training in 1 as 5.28. The sparsity level in 2 is 2. The number of dictionary atoms is 128. The dictionary is obtained by training with 20 OCT images, which are high resolution and high SNR. As a demonstration in the following experiments, we select 10 training images from coronary artery sample and 10 images from onion sample, and the number of patches extracted from the 20 training images is 11 670 550. Specifically, the volumes where we select the training images are different from the test volumes, so that the dictionary is generic to decompose a large variety of images from distinct volumes. Training process takes 20 iterations, using 8 *by* 8 as patch size. The threshold for saturation detection in Section 2.2 is 99 975.6, which is full well capacity measured from the spectrum in the experimental SDOCT system.

#### 3.2.2 Synthetic Artifacts:

**Fig. 4.**
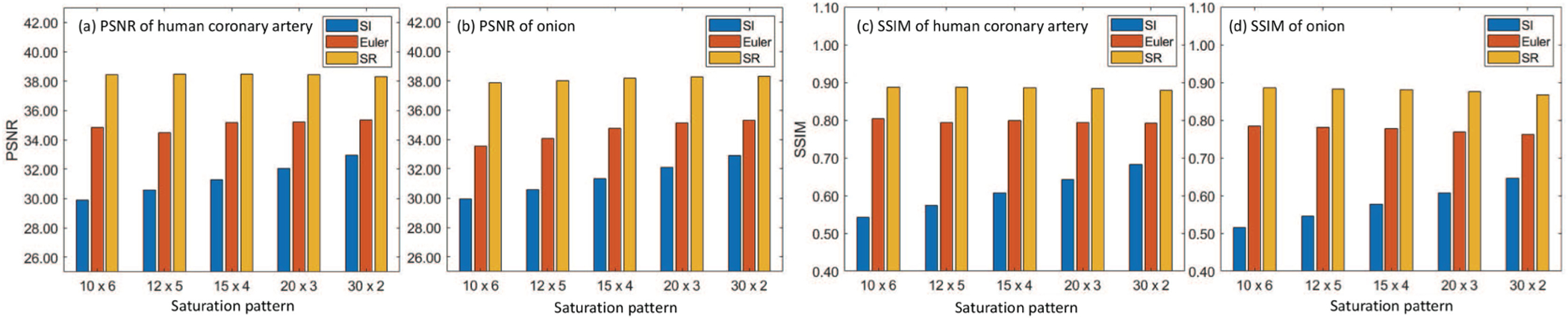
Comparison of (a), (b) PSNR and (c), (d) SSIM on human coronary artery and onion tissue over different methods. *x* × *y* means generating x pairs of saturation, with each pair taking y consecutive A-lines.

We first generate synthetic artifact mask to quantitatively evaluate the inpainting performance. To evaluate the performance of the proposed method and the comparison methods, we generate random masks as detected saturation and overlayed the masks with experimental images. Each random mask contains 60 saturated A-lines. For each experimental image, we apply 5 different patterns of *x* × *y*, which represents *x* pairs of *y* consecutive saturated A-lines. To include a wide range of saturation levels, we setup the patterns from sparse saturation (30 × 2) to dense saturation (10 × 6).We use the peak-signal-to-noise (PSNR) to evaluate the intensity difference and structural similarity index (SSIM) to quantify the perceptual difference between reference and output image. The reference image is obtained by averaging consecutive images (*N* = 5). The comparison methods are SI and Euler’s elastica method [12]. The synthetic experimental images are from coronary (*N*_1_=20) and onion (*N*_2_=20), which are not seen in training dictionary. The comparison of PSNR and SSIM within saturated region is shown in Fig. 4. We observe a decreasing trend of PSNR in SI and Euler’s elastica when saturation gets denser but not the proposed method. The performance of SI drops drastically (~3 dB) compared to our method (~0.5 dB). Euler’s elastica has relatively small drop (1.5 dB) but generally 3~4 dB lower than our method. Same decreasing trend of SSIM can be observed in SI when saturation gets denser. SI’s performance drops by ~0.15, Euler’s elastica’s performance is stable but generally 0.1 lower than our method. The proposed method is more robust to the SI and Euler’s elastica.

**Fig. 5.**
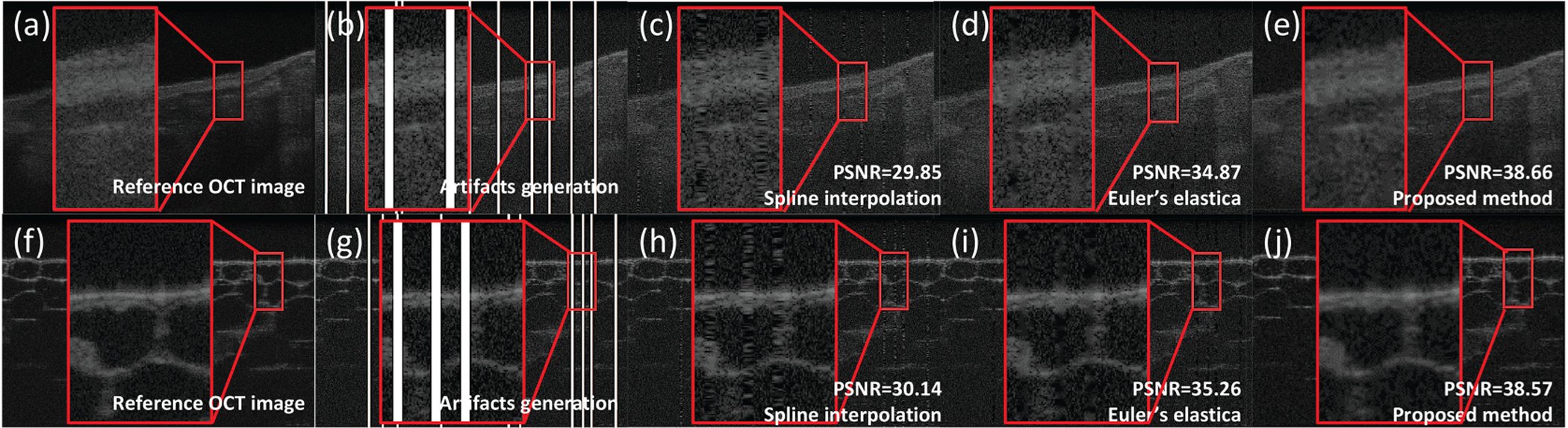
Experimental results with synthetic saturation artifacts: (a), (f) Reference OCT images; (b), (g) Artifacts generation; (c), (h) Output of SI; (d), (i) Output of Euler’s elastica; (e), (j) Output of the proposed method. Figs. (a)–(e) are from coronary artery sample and Figs. (f)–(j) are from onion sample.

Fig. 5 shows a visual comparison among methods for inpainting coronary artery image and onion image. The output of the proposed method (Fig. 5 e, j) is more homogeneous than the SI and Euler’s elastica. In Fig. 5(c) and Fig. 5(h), the images inpainted by SI show severe discontinuity. Euler’s elastica generates a smooth transition but results in local blur near the generated artifacts. The proposed method can effectively correct the artifacts, reduce the noise, and maintain the texture information compared to the reference image.

#### 3.2.3 Real Artifacts:

**Fig. 6.**
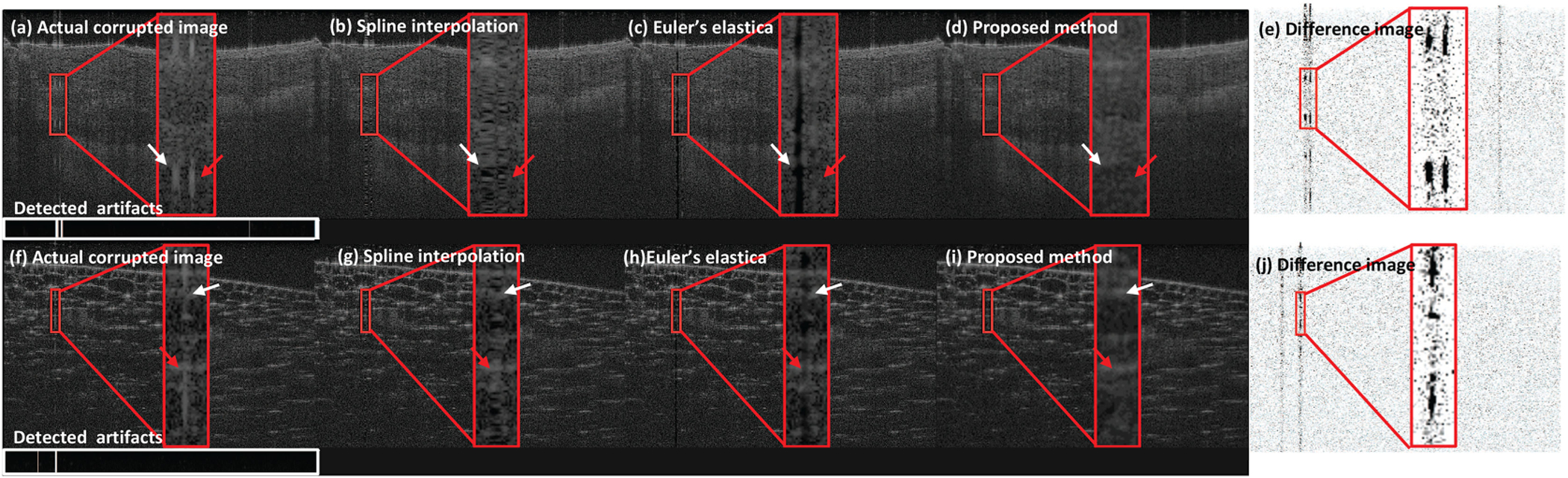
Experimental results with real saturation artifacts: (a), (f) Original image with artifact detection results; (b), (g) Output of SI; (c), (h) Output of Euler’s elastica; (d), (i) Output of our method; (e), (j) Difference image between the original image and output of our method (with inverted contrast for better visualization). Figs. (a)–(e) are from coronary artery sample and Figs. (f)–(j) are from onion sample. White boxes in (a), (f) are results from artifact detection on A-line level (black: no saturation, white: detected saturation).

The image with real artifacts is processed with the artifact detection in section 2.2. The image aligned with the column-wise mask of detected saturation is fed into patch-wise processing. Fig. 6 demonstrates a visual comparison for inpainting images with real saturation artifacts. The horizontal bars shown below the original OCT image (Fig. 6(a) and Fig. 6(f)) are detected saturation masks. Our method successfully detected the saturation artifacts in the original image. As can be seen in the coronary artery (Fig. 6 (a)–(d), ripple effect from SI turns obvious when the saturation is denser (white arrow in Fig. 6(b)). Euler’s elastica yields smooth transition when the artifacts are mild (red arrow in Fig. 6(c)) but distinct vertical line when handling dense saturation artifacts (white arrow in Fig. 6(c)). This is because Euler’s elastica relies upon curvature information nearby to inpaint the saturated region. However, in the dark region, little curvature information can be used, leading to a vertical line that cannot be optimized. A similar observation is made for the onion sample (Fig. 6 (f)–(i). Such ripple effect and distinct vertical line impact the visual quality of the OCT image. On the contrary, the proposed method produces high quality OCT image after inpainting the artifacts region competently. The difference images (Fig. 6(e) and Fig. 6(j)) are computed between the original image and the output of our method, and shown with inverted contrast for better visualization. As can be seen in Fig. 6(e) and Fig. 6(j), speckle noises are filtered as well. In the proposed method (Fig. 6(d) and Fig. 6(i)), saturation artifacts are suppressed compared to the actual corrupted image (Fig. 6(a) and Fig. 6(f)) as evidenced in the red inset region.

#### 3.2.4 Generalization experiments:

**Fig. 7.**
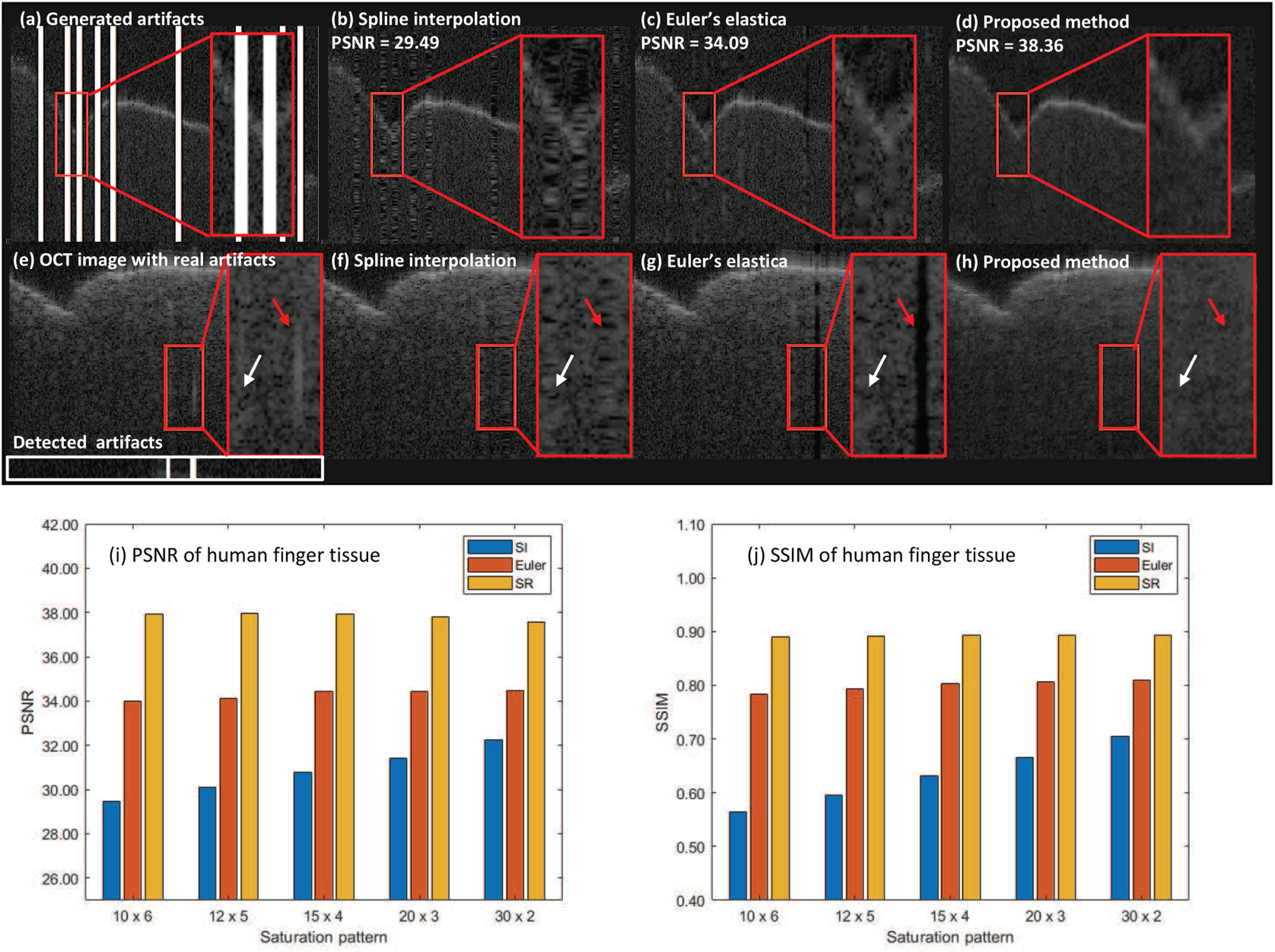
(a)–(d) Synthetic artifacts experiment on finger tissue. (e)–(h) Real artifacts experiment on finger tissue. Comparison of PSNR (i) and SSIM (j) over different methods. (a)Reference OCT images; (e) Image with real artifacts; (b), (f) Output of SI; (c), (g) Output of Euler’s elastica; (d), (h) Output of the proposed method. White box in (e) is result from artifact detection on A-line level (black: no saturation, white: detected saturation).

To evaluate the feasibility of the proposed method to other tissues, we adopt finger tissue, the images of which are not seen in training data, and generated random masks as detected saturation to quantitatively measure the performance. The comparison of PSNR in Fig. 7(i) shows that proposed method is robust to saturation levels than SI. The large drop (2.76 dB) can be observed in SI while our method even performs better when the saturation turns denser. The PSNR of Euler’s elastica is lower than our method by 3.08~3.91 dB. The SSIM result (Fig. 7(j)) indicates that the large drop of SI is ~0.15, while our method drops by ~0.002. Euler’s elastica is generally ~0.1 lower than our method. In terms of visual quality, we apply the artifact detection and inpainting algorithm on images of finger tissue with real artifacts. Ripple effects in Fig. 7(f) and distinct vertical line in Fig. 7(g) turns obvious as the saturation gets denser (red arrows). The outcome of purposed method in Fig. 7(h) yields a smooth transition over the edge of saturation artifacts and better visual quality. The experiments demonstrate that our method can be generalized to other tissue type, which is not included in training.

## 4. Discussion

In this paper, we propose a novel method using dictionary-based sparse representation to correct the saturation artifacts in OCT image. The experiment on synthetic artifacts and real artifacts demonstrates that the proposed method effectively corrects saturation artifacts. We are able to localize the saturation artifacts on A-line level and correct the saturation artifacts from sparse to dense with high quality. Furthermore, we adopt a general dictionary so that it could be utilized to inpaint tissue samples which are not initially included in training data.

An important application of this work is to better the accuracy of segmentation. In clinic diagnosis, accurate estimation of tissue boundaries is crucial to determine the quantitative parameter for treatment [24]. With the existence of saturation pixels, the pixel information needed to estimate boundaries is blurry. The correction of saturation artifacts could yield better estimated pixels and thus produce better boundary estimation for segmentation.

There are two major limitations of our study. First, the performance is limited by memory. If the whole patch is corrupted, there is not enough unsaturated pixel to recover corrupted pixel information in Eq. 3. Therefore, the capability to recover N consecutive saturated A-lines is limited by the size of patch. However, large size of patch requires large amount of memory in dictionary training process and sparse-coding process. Second, the computational time for a single B-scan is approximately 90 seconds. Such computational time is not sufficient to achieve real-time image acquisition. The implementation of GPU or distributed computing may contribute to accelerate the computation. In addition, our future work will also include to the application of this saturation correction method to boost the performance of OCT segmentation.

## 5. Conclusion

We demonstrate the feasibility of localizing and inpainting saturation artifacts in OCT images. We propose an artifact correction method via inpainting. The generic dictionary can be utilized to decompose images of tissue sample which is not included in training datasets. We have successfully localized saturated A-lines and achieved a better performance than SI and Euler’s elastica in inpainting saturated regions. Our method produces OCT images with high visual quality as a result of inpainting based on dictionary-based sparse representation scheme.
